# Formulation and evaluation of injectable dextran sulfate sodium nanoparticles as a potent antibacterial agent

**DOI:** 10.1038/s41598-021-89330-0

**Published:** 2021-05-10

**Authors:** Osama A. Madkhali, Sivakumar Sivagurunathan Moni, Muhammad H. Sultan, Haitham A. Bukhary, Mohammed Ghazwani, Nabil A. Alhakamy, Abdulkarim M. Meraya, Saeed Alshahrani, Saad Saeed Alqahtani, Mohammed Ali Bakkari, M. Intakhab Alam, Mohamed Eltaib Elmobark

**Affiliations:** 1 Department of Pharmaceutics, College of Pharmacy, Jazan University, P.O. Box 114, Jazan, Postal Code 45142 Saudi Arabia; 2 Pharmacy Practice Research Unit, Clinical Pharmacy Department, College of Pharmacy, Jazan University, Jazan, Saudi Arabia; 3 Pharmacology and Toxicology Department, College of Pharmacy, Jazan University, Jazan, Saudi Arabia; 4 Department of Pharmaceutics, College of Pharmacy, Umm Al-Qura University, Makkah, 24381 Saudi Arabia; 5 Department of Pharmaceutics, College of Pharmacy, King Khalid University, Abha, Saudi Arabia; 6 Department of Pharmaceutics, Faculty of Pharmacy, King Abdulaziz University, Jeddah, Saudi Arabia; 7 Center of Excellence for Drug Research and Pharmaceutical Industries, King Abudlaziz, University, Jeddah, Saudi Arabia

**Keywords:** Nanomedicine, Drug delivery

## Abstract

The purpose of this study was to develop a novel nano antibacterial formulation of dextran sulfate sodium polymer. The dextran sulfate sodium (DSS) nanoparticles were formulated with gelation technique. The nanoparticles exhibited significant physicochemical and effective antibacterial properties, with zeta potential of − 35.2 mV, particle size of 69.3 z d nm, polydispersity index of 0.6, and percentage polydispersity of 77.8. The DSS nanoparticles were stable up to 102 °C. Differential scanning calorimetry revealed an endothermic peak at 165.77 °C in 12.46 min, while XRD analysis at 2*θ* depicted various peaks at 21.56°, 33.37°, 38.73°, 47.17°, 52.96°, and 58.42°, indicating discrete nanoparticle formation. Antibacterial studies showed that the DSS nanoparticles were effective against Gram-positive and Gram-negative bacteria. The minimum inhibitory concentrations of DSS nanoparticles for *Bacillus subtilis* (*B. subtilis*), *Staphylococcus aureus* (*S. aureus*), *Streptococcus pyogenes* (*S. pyogenes*), *Escherichia coli* (*E. coli*), *Pseudomonas aeruginosa* (*P. aeruginosa*), *Klebsiella pneumoniae* (*K. pneumoniae*) *and Proteus vulgaris* (*P. vulgaris*) were 150, 200, 250, 150, 200, 250, 250 µg/mL, respectively*.* The antibacterial effects of DSS nanoparticles were in the order *E. coli* (26 ± 1.2 mm) at 150 µg/mL > *S. pyogenes* (24.6 ± 0.8 mm) at 250 µg/mL > *B. subtilis* (23.5 ± 2 mm) at 150 µg/mL > *K. pneumoniae* (22 ± 2 mm) at 250 µg/mL > *P. aeruginosa* (21.8 ± 1 mm) at 200 µg/mL > *S. aureus* (20.8 ± 1 mm) at 200 µg/mL > *P. vulgaris* (20.5 ± 0.9 mm) at 250 µg/mL. These results demonstrate the antibacterial potency of DSS injectable nanoparticles.

## Introduction

The treatment of infectious diseases caused by bacterial organisms poses a lot of problems, especially nosocomial infection^1–4^. Bacterial resistance and multiple antibiotic resistance are emerging global health care problems caused by treatment failure, leading to morbidity and mortality^5–8^. Bacterial resistance to antibiotics/antibacterials is a significant challenge for researchers in their quest to overcome poor therapeutic outcomes caused by bacterial expression of resistant factors. Irrational use of antibiotics is a major factor for the emergence of bacterial resistance worldwide, especially in developing countries, due to lack of adequate knowledge of appropriate use of these drugs^9^. Antibacterial resistance is due to mutation in the plasmid gene that encodes the resistance (R) factor, which causes changes in membrane permeability, lytic enzymes, and efflux pumps^4, 10^. Biodegradable and biocompatible nanoparticles composed of synthetic or natural polymers offer a better delivery system, thereby decreasing drug-related toxicity and drug resistance, while reducing the drug dose used by enhancing bioavailability^11^.

Researchers across the world have continued to develop novel antibiotics for combating infections. Unfortunately, these newer molecules do not prevent drug resistance. The need to deliver antibiotics at the cellular level so as to overcome these resistant channels has become a challenge to researchers. This has prompted pharmaceutical scientists to develop new drug delivery systems for overcoming multiple drug resistance. One of such approaches is the use of nanotechnology to facilitate effective regulation of drug delivery to an infection site, thereby preventing toxicity. Dextran sulfate sodium, a natural polymer of α-1,6-d-glucopyranose, is hydrophilic, biodegradable and biocompatible^12, 13^. The biological properties of dextran sulfate have been reported to show anti-coagulant, antiviral, and lowering the cholesterol level^14–17^.

An earlier study showed the development of dextran sulfate nanoparticles as a targeted delivery system for methotrexate, an immunosuppressive agent used for managing rheumatoid arthritis^18^. Moreover, chemically modified dextran has been reported to exert antibacterial property, specifically against *S. aureus*^19^. A dextran-based nano bio-composite membrane containing essential oils from herbs has been developed as a topical formulation^20^. A recent study suggested that sanguinarine-containing hydrogels prepared using dextran–hyaluronic acid enhanced healing of burn wounds^21^. The present study was aimed at developing injectable dextran sulfate nanoparticle formulation for use as a potential antibacterial agent.

## Materials and methods

### Materials

Dextran sulfate (sodium salt, MW = 500,000) and sodium tripolyphosphate (MW = 367.86) were purchased from Santa Cruz Biotechnology, Inc, Dallas, USA. Bacteriological media and other chemicals and solvents were purchased from Scharlau, Spain. All the materials used in this research were supplied by Ejadah Medical Supplies Est, Riyadh, Saudi Arabia.

### Formulation of DSS nanoparticles

A range of methods for preparation of dextran nanoparticles was used in this study^22^. The nanoparticles were prepared through a gelation technique, with dextran sulfate sodium (DSS) as the polymeric carrier, while tripolyphosphate (TPP) was used as a stabilizer. Three different formulations (Batch 1, 2, 3) were optimized by varying the concentrations of DSS and TPP. The DSS:TPP ratio was varied with individual formulations: the proportions were 1:10, 2:5, and 1:5. The solution was kept on a hot plate and stirred with a magnetic bead at a constant speed (2000 rpm) for 90 min. The temperature of the reaction mixture was maintained at 60 °C, and 2% TPP (w:v) was added dropwise at pre-determined time intervals during the preparation of the nanoparticle formulations. Each mixture was stirred with a magnetic stirrer for 60 min at 60 °C. During the mixing process, sonication was performed at pre-determined time interval for 2 min at 100% amplification using a laboratory probe sonicator, CPX ultrasonic processor (Cole Parmer Instruments Co, USA). Finally, the mixture was filtered through Millex-GV Syringe Filter Unit, 0.2 µm, PVDF, Merck KGaA, Darmstadt, Germany. The filtrate was collected in a sterile glass screw cap tube and stored in a refrigerator at 4 °C prior to use in subsequent experiments. Batch 3 was determined to be the best batch when compared to other batches.

### Lyophilization process

Lyophilization or freeze-drying is a technique that improves the stability of nanoparticles. Lyophilization was achieved using Millrock BT85 tabletop freeze dryer (Millrock Technology, USA). A 5% w/v mannitol solution was mixed with the DSS nanoparticle reaction mixture at a 1:1 volume ratio in a glass flask. The mixture was kept in a deep freezer at − 80 °C for 24 h. Thereafter, the frozen DSS nanoparticles were fixed in lyophilizing pipelines, with the knob opened for induction of vacuum. The vacuum was maintained at 3000 pascals, while temperature was maintained at − 84 °C. The lyophilization process was continued for 24 h, after which the lyophilized nanoparticle powder was removed from the glass flask, pooled, and stored in a refrigerator at 4 °C for further studies.

### Determination of physical characterization of DSS nanoparticles

#### Dynamic light scattering (DLS) analysis

Surface charge, particle size, and PDI are essential parameters used for physical characterization of nanoparticles in an injectable dosage form. Zeta potential (ZP) analysis was carried out to determine the surface charge on the nanoparticles in an injectable colloidal system. The nanoscale particle size (NS) of the DSS nanoparticles and their polydispersity index (PDI) in the injectable colloidal system were determined using dynamic light scattering (DLS) technique. In this study, ZP, NS, and PDI were determined using a Nano-ZS Zetasizer (Malvern Instruments, UK). The formulated injectable liquid nanoparticles filtered through Millex-GV Syringe Filter Unit, 0.2 µm, PVDF, Merck KGaA, Darmstadt, Germany. The liquid filtrate was placed in a folded capillary cell with no air bubbles and placed in the instrument holder. The colloidal liquid injectable formulation was tested using a standard procedure in accordance with the manual guidelines provided by Malvern Instruments, UK.

### Determination of morphological features of DSS nanoparticles

#### Scanning electron microscopy

The morphological features of the lyophilized DSS nanoparticles were studied with a high-resolution scanning electron microscope (SEM) using JEOL JSM 6360 (JEOL USA, Inc, Japan). A powder sample was placed on metal stubs and coated with gold–palladium to a thickness of 200–300 Å under reduced pressure. The image of DSS nanoparticles was observed at various magnifications^23^.

#### Transmission electron microscopy

Transmission Electron Microscopy (TEM) is a technique that results in very high resolution of images of nanoparticles. A powder sample of lyophilized DSS nanoparticles was characterized using JEOL JEM-1011 transmission electron microscope (JEOL USA, Inc, Japan). The TEM grid was prepared by placing the sample on a carbon-coated grid, and the instrument was operated at 200 kV. The TEM procedure was carried out in line with the method reported earlier^24^.

### Differential scanning calorimetry (DSC) analysis of DSS nanoparticles

Differential scanning calorimetry is a thermal analytical technique used to determine enthalpy changes due to changes in physicochemical properties of powdered samples. In this study, DSC analysis of the DSS nanoparticles was performed with DSC 60 (Shimadzu, Japan) according to the method developed as reported earlier^25^. The powder nanoparticle sample was placed in non-hermetically sealed aluminium pans. The temperature was raised from 30 to 350 °C at a heating rate of 10 °C min^−1^, and atmospheric airflow was maintained at 10 mL min^−1^.

### X-ray diffraction (XRD) analysis of DSS nanoparticles

The crystalline structure of the DSS nanoparticles was determined with X-ray diffraction (XRD) analysis of the powder sample, using the method reported earlier^26^. Crystalline structure is used as an index of purity of DSS nanoparticles. The powder sample was subjected to XRD using a Unisantis XMD 300 X-ray powder diffractometer (Unisantis Europe GmbH, Germany). The XRD diffractograms were obtained at 2*θ* in the range 2°–50° using Cu K α radiation of incident beam (*λ* = 1.5418 Å) at a voltage of 45 kV and a current of 0.8 mA. A scanning range of 2*θ*/*θ* was selected, and scanning speed of 10 min^−1^ was employed.

### Antibacterial studies of DSS nanoparticles

#### Standardization of bacterial culture

In this study, laboratory bacterial cultures of the human pathogenic bacteria *S. aureus*, *S. pyogenes, B. subtilis, E. coli, P. aeruginosa, K. pneumoniae* and *P. vulgaris* were used. The bacterial cultures were prepared by subculturing from stock cultures in nutrient broth, and incubating in a bacteriological incubator at 37 °C for 24 h. The bacterial cultures were then diluted in Millipore water (from 10^−1^ to 10^−7^ dilution) in sterilized nutrient broth. The potential viability of each bacterial organism was measured by determination of colony-forming unit in 1 mL (CFU/mL). A standardized culture was utilized to determine the minimum inhibitory concentration of the DSS nanoparticles for each bacterial culture, as well as the spectrum of antibacterial activity of the nanoparticles.

#### Minimum inhibitory concentration (MIC) study

The MIC of the DSS nanoparticles for the tested bacterial organisms was determined with broth dilution method according to the standard protocol established by the Clinical and Laboratory Standards Institute^27^.

#### Agar well diffusion assay

The spectrum of antibacterial activity of DSS nanoparticles (test sample) and that of standard drug ciprofloxacin (50 µg/mL) against the selected human pathogenic organisms were obtained using the agar well diffusion technique^28^. The test for evaluating the antibacterial activity was performed using Muller Hinton (MH) agar plates. Standardized cultures were inoculated twice on MH agar plates using the spread plate technique. The agar well diffusion technique was carried out by punching holes on inoculated MH agar plates (for both test and standard samples) using standard sterile stainless-steel borers. The 100 µL of 1% w/v of test samples and standard were placed aseptically in different wells and allowed to diffuse for 10 min. Then, the plates were incubated at 37 °C for 24 h, and the antibacterial spectrum was determined through the appearance of inhibitory zones after 24 h of incubation. The diameter of the zone of inhibition was used as an index of the spectrum of activity.

### Statistical analysis

Each experiment was performed six times (n = 6), and the data were subjected to one-way analysis of variance (ANOVA). The levels of statistical significance were *p* < 0.001 (extremely significant) and *p* < 0.01 (significant). Statistical analyses were done using Prism 9 Graph Pad Instat software system, USA. Values for the test samples were compared with values for the standard drug using Dunnet's post hoc test.

## Results and discussion

**Figure 1:**
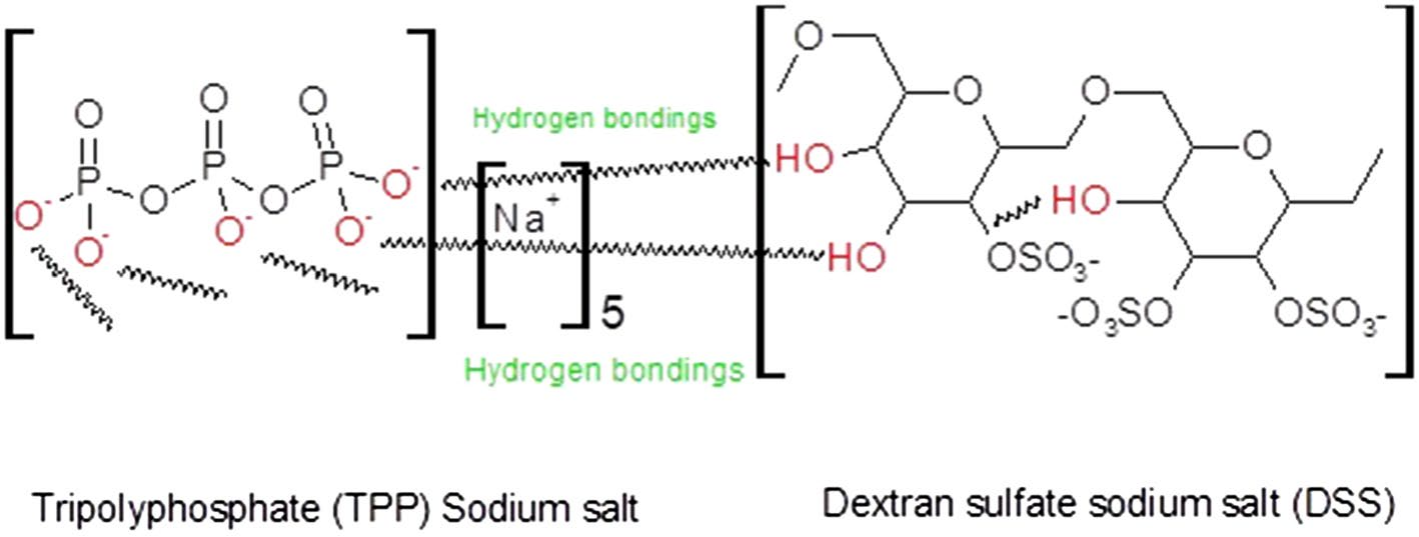
Possible chemical reaction between sodium salt of tripolyphosphate and dextran sulfate sodium salt.

Nanoparticle formulation is a very important technology that can be used to solve problems of drug resistance that arise in conventional antibiotic therapy^29, 30^. The advantage of nanoparticles lies in their use for eliciting intracellular effects in specific cells. The sodium salt of dextran is an anhydrous and hydrophilic glucose polymer composed of approximately 95% alpha-D-glucose linkages. Sodium salt of TPP, a penta-anionic compound, was used as cross-linker and stabilizer. The gelation technique was used to develop DSS nanoparticles by linking DSS and TPP with hydrogen bond formation . The formation of hydrogen bonds between DSS and TPP is shown in (Fig. 1). The chemical reaction between dextran sulfate and TPP can be attributed to the formation of hydrogen bonds between the -OH groups of dextran sulfate and the oxygen atoms of TPP. Besides, there is a possibility of formation of phosphate ester with dextran OH groups through esterification process^31^. An earlier report suggested that dextran phosphates esterification can be achieved by the interaction of hydroxyl groups and phosphate groups that led to the formation of mono- and diesters. According to a previous study, dextran phosphates were esterified by the interaction of hydroxyl and phosphate groups, which results in the formation of mono- and diesters^32^. Physical and chemical crosslinking with hydrogen bonds and electrostatic interactions have been demonstrated in designed biodegradable hydrogels^33^. In this study, the sodium salt of DSS reacted with sodium salt of TPP under mild heat, resulting in development of suitable nanoparticles formulated by varying the ratio of polymer to cross-linker.

### Physical characterization of DSS nanoparticles

**Figure 2:**
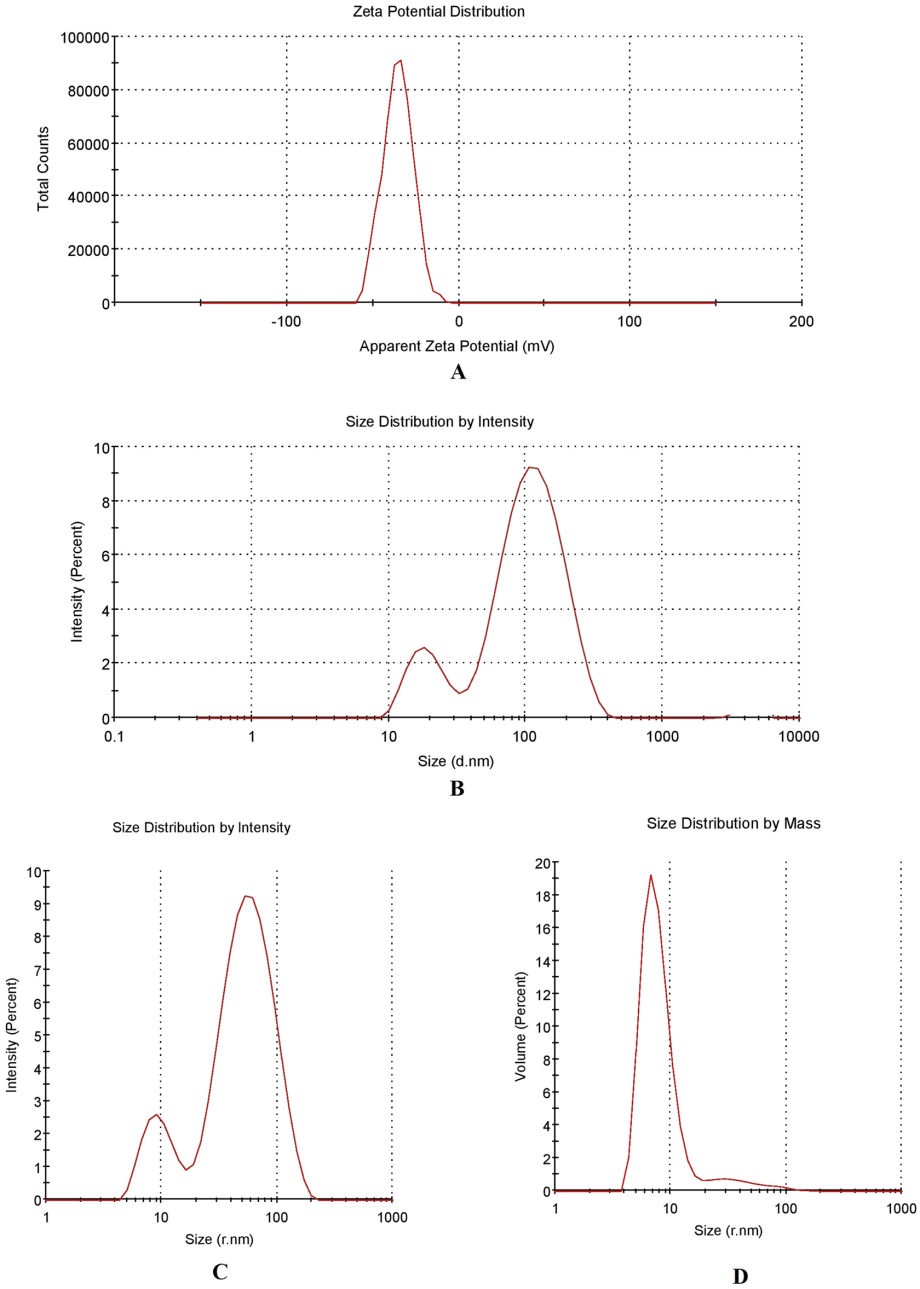
Physical characterization of 1% w/v of dextran sulfate sodium (DSS) nanoparticles of batch 3. (**A**) Zetapotential graph of DSS nanoparticle. (**B**) Nano size (z d nm) distribution of DSS nanoparticles through particle intensity. (**C**) Size (r nm) distribution by intensity analysis of DSS nanoparticles. (**D**) Size (r nm) distribution analysis by mass.

**Figure 3:**
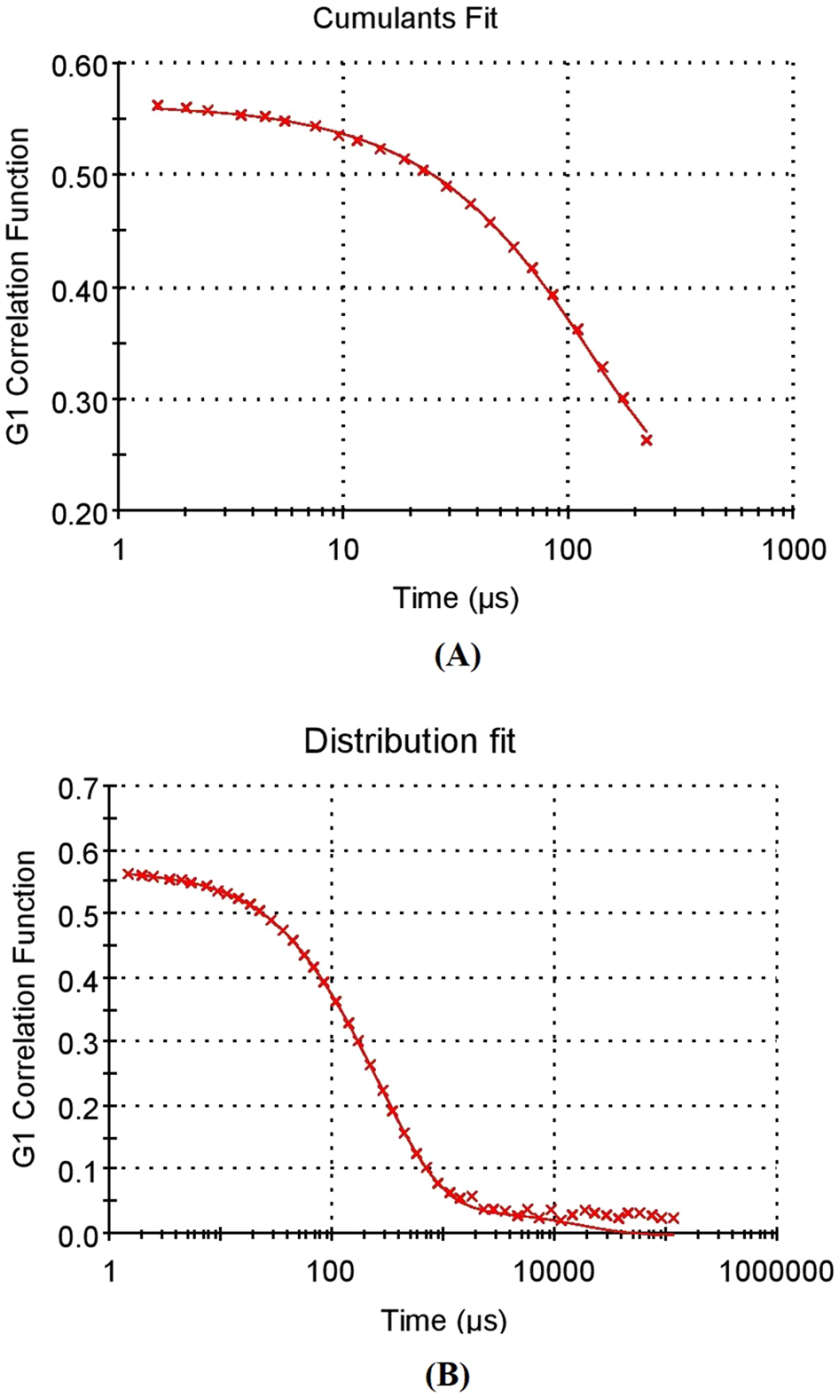
Physical characterization of 1% w/v of dextran sulfate sodium (DSS) nanoparticles of batch 3. (**A**) Cumulative fit analysis of DSS nanoparticle. (**B**) Size distribution fit analysis of DSS nanoparticles.

The lyophilized DSS nanoparticles were in the form of free-flowing powder. The physical characteristics of various batches of screened DSS nanoparticles are depicted in Table 1. The results demonstrate the influence of ratio of polymer-to-crosslinker on nanoparticle characteristics. DSS nanoparticles in batch 3 showed the best physical characteristics, and were the most successful formulation, when compared to the other batches. The potential of DSS nanoparticles is reflected in their physical characteristics. In this study, the injectable DSS nanoparticles exhibited good physical characteristics. Figure 2 shows the ZP of the DSS nanoparticles. Nanoparticles developed in batch 3 had unique ZP with a single peak (Fig. 2A). Interestingly, the ZP of the DSS nanoparticles was − 35.2 mV when polymer: cross-linker ratio was 1:5. This accounts for the development of highly stable nanoparticles. The PDI value was 0.6, while the % polydispersity was 77.8, indicating the formation of a homogenous system in batch 3, when compared to the other batches. In batch 2, the DSS nanoparticles had PDI of 0.538, and the % polydispersity was 73. However, the DSS nanoparticles exhibited ZP of − 21.6 mV, which indicated that they had lower stability than batch 3 DSS nanoparticles. The intensity peak value of batch 3 nanoparticle size is shown in Fig. 2B–D representing a bimodal size distribution. The major peak with 69.3 z d nm indicated the maximum particles size (Fig. 2B. The particle size (in terms of radius) was in the range of 52–82 nm (Fig. 2C). The particle size characterization also showed that batch 3 DSS nanoparticles were better than DSS nanoparticles from other batches. The size distribution of DSS nanoparticles based on % mass (in r nm) of a colloidal system is shown in Fig. 2D. With a % mass (r nm) value of 90 which indicated uniform size distribution in the colloidal nanoparticle system, batch 3 DSS nanoparticles were the best formulation. The percentage of size distribution in batches 1 and 2 DSS nanoparticles were 44 and 49%, respectively, indicating particle aggregation and non-suitability for application in injectable dosage forms. Cumulative fit analysis of particle distribution is shown in Fig. 3A. The DSS nanoparticles showed good quality in a colloidal dispersive system, with 100% linearity. The results indicated that batch 3 formulation was of the highest quality. Figure 3B shows the size distribution fit, with 10% variation. However, the quality of the distribution fit was better than that indicated on the instrument, demonstrating that the formulations were successfully produced. The mobility of DSS nanoparticles is a significant factor in particle dispersion in injectable dosage forms. In this study, high mobility was observed in batch 3, when compared to DSS nanoparticles from the other batches (Table 1).

**Table 1:** Physical characterization of dextran sulfate sodium (DSS) nanoparticles.

Batches	Polymer	Cross linker	Polymer: crosslinker ratio	Zeta (mV)	PDI	% Poly dispersity	Particle size in mass (r nm)	% Mass (r nm)	Particle size (z d nm)	Mobility (µm cm/Vs)	Conductivity (mS/cm)
Batch 1	DSS	TPP	1:10	− 27.7	1	100	5.05–30.74	49.32	57.96	− 2.170	10.5
Batch 2	DSS	TPP	2:5	− 21.6	0.538	73.74	3.765–5.5	44.73	13.90	− 1.277	3.77
Batch 3	DSS	TPP	1:5	− 35.2	0.605	77.8	52–82	90	69.3	− 3.857	3.78

*DSS* dextran sulphate sodium, *TPP* tripolyphosphate, *PDI* poly dispersity index.

The pharmacokinetic profiles of nanoparticles depend on the surface charge and the size, which are two factors that influence biodistribution^34^. The ZP value of − 35 mV suggests passive targeting of bacterial cells. Nanoparticle mobility in a colloidal injectable system is also an essential factor that influences particle conductivity. Particle sizes less than 20 nm have high mobilities in the colloidal system and influence particle surface charge^35^. Interestingly, in the present study, the particle size and size distribution were 13.90 z d nm and 3–5 r nm, respectively, but particle surface charge and mobility were not developed, probably due to the non-homogenous system with % mass of 44 r nm. In contrast, batch 3 DSS nanoparticles were homogenous colloids with % mass of 90 r nm. Enhanced permeability and retention (EPR) effect, an essential factor in the targeting of bacterial cells, is influenced by nanoparticle size^36^. Bacteria have variable pore sizes. A previous study reported that *S. aureus* had a pore ranging in size from 50 to 500 Å, and a diameter in the range of 5–50 nm^37^. The DSS nanoparticles developed in batch 3 had a particle size of 69.3 z d nm and radius in the range of 52–82 r nm, indicating easy passive diffusion of the DSS nanoparticles through the bacterial cell pores. Therefore, passive targeting of DSS nanoparticles to bacterial cells can be achieved through formulation of nanoparticles with optimum size. The therapeutic significance of DSS nanoparticle can be achieved through a cellular uptake process dictated by the size of the particles, resulting in clinical effectiveness.

### Morphological analysis of DSS nanoparticles

**Figure 4:**
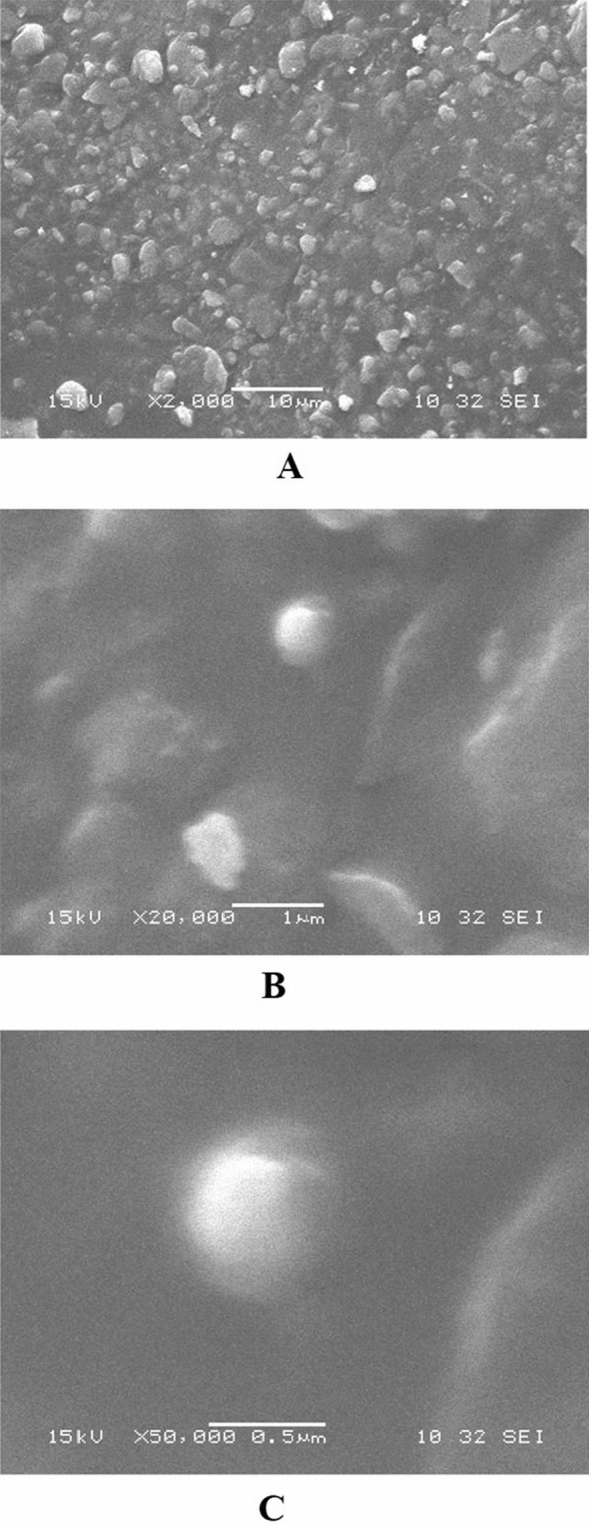
Scanning electron micrograph of dextran sulfate sodium (DSS) nanoparticles of batch 3. (**A**) DSS nanoparticle at × 2000 magnification. (**B**) DSS nanoparticles at × 10,000 magnification. (**C**) DSS nanoparticles at × 50,000 magnification.

**Figure 5:**
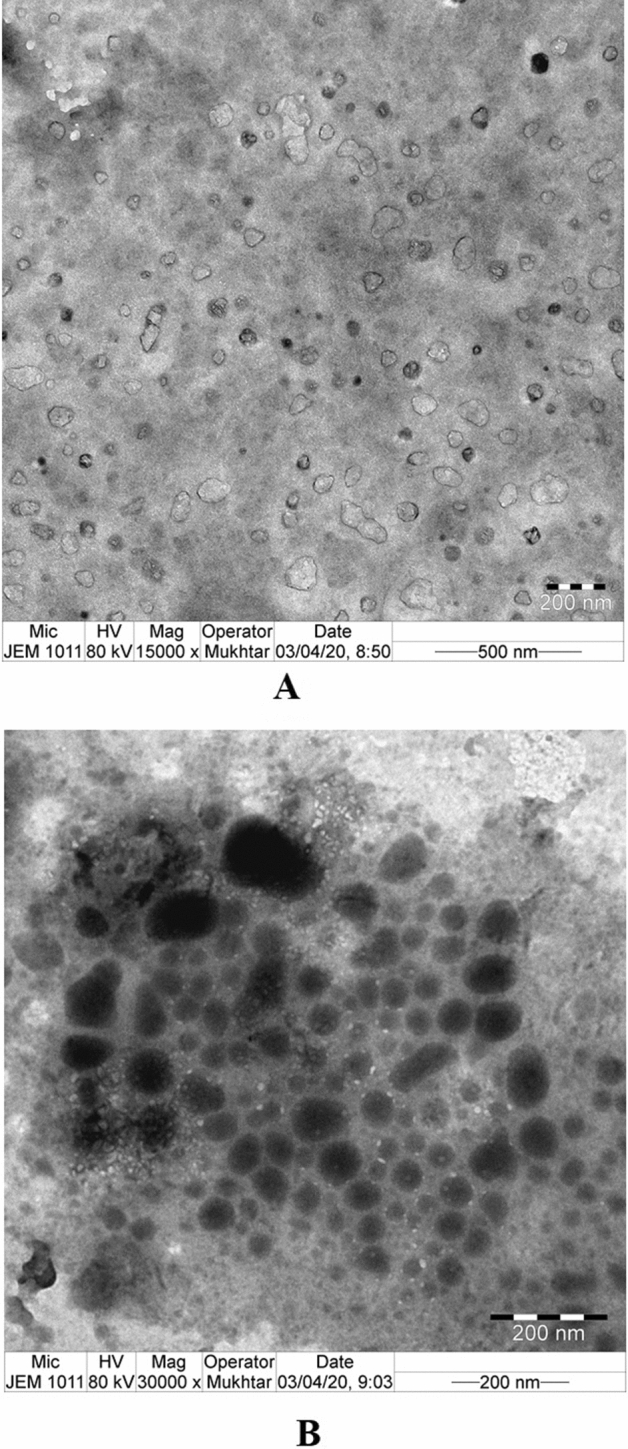
Transmission electron micrograph of dextran sulfate sodium (DSS) of batch 3 nanoparticles. (**A**) DSS nanoparticle at × 15,000 magnification. (**B**) DSS nanoparticles at × 30,000 magnification.

The results of SEM analysis of DSS nanoparticles are presented in Fig. 4. The DSS nanoparticles are shown at 2000 × magnification, revealing a more or less spherical particles at high image resolution (Fig. 4A). However, few particles are clumped and crystalline in morphology. At 20,000 × magnification, the spherical particles had rough surfaces (Fig. 4B). Interestingly, at 50,000 × magnification, discrete, spherical particles with rough surfaces were observed (Fig. 4C). It is note worthy that the SEM analysis was showing particle aggregation which might be due to lyophilization process. Recently, it was reported that nanoparticles produced by cross-linking of dextran sulfate with sodium selenite nanoparticles showed crystalline morphology with rough surfaces but failed in physical characterization^22^. The results TEM of DSS nanoparticles are presented in Fig. 5A and B. At 15.000 × magnification, the DSS nanoparticles were more or less spherical in shape, with rough surfaces, and the particles were discrete. Interestingly, at 30,000 × magnification, crowded particles were observed with spherical and elongated shapes, as well as ruptured particles. The particles were of non-uniform sizes, ranging from 30 to 70 nm in diameter. Additionally, we were able to observe differences in particle size distributions using SEM and TEM analysis. An earlier report showed that the size of silver nanoparticles was smaller in TEM analysis than the size observed using DLS technique^26^. In contrast to the present study, there was consistency in size of DSS nanoparticles between TEM and DLS analyses.

### Differential scanning calorimetry analysis of DSS nanoparticles

**Figure 6:**
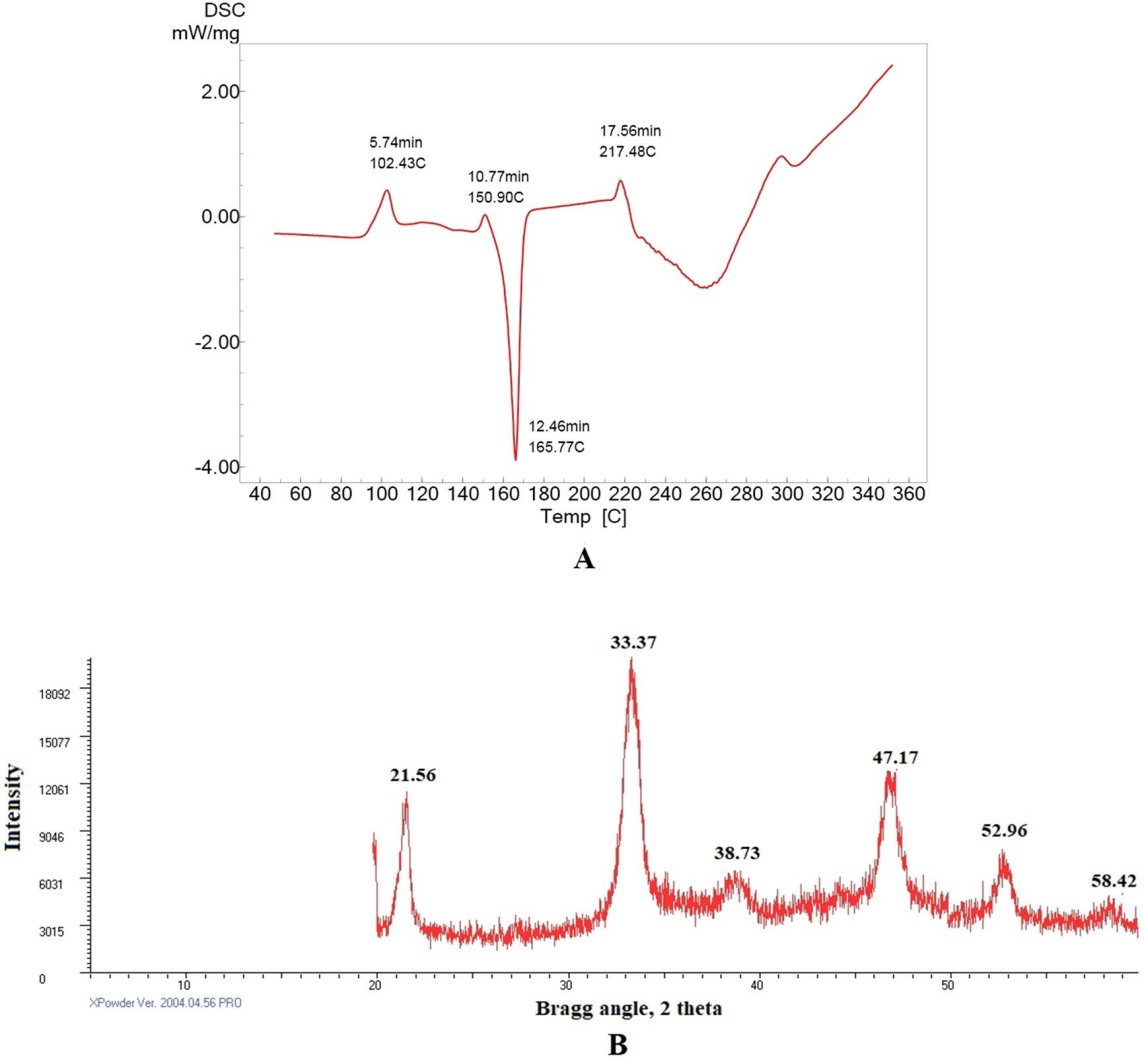
(**A**) First heating cycle of differential scanning calorimetry analysis of DSS nanoparticles, at a heating rate of 10 °C min^−1^, and atmospheric airflow was maintained at 10 mL min^−1^. (**B**) XRD analysis of DSS nanoparticles, the diffractogram was obtained at 2*θ* in the range 2°–50°.

This technique (DSC) measures changes in thermodynamic parameters such as enthalpy, entropy, and heat capacity in nanoparticles due to physical factors, chemical reactions, and phase transitions. The thermal degradation property of DSS nanoparticles was investigated using DSC analysis over a temperature range of 40–360 °C (Fig. 6A). A molecular weight change was observed from 102.82 to 165.77 °C. The DSS nanoparticles were stable up to 102 °C. Similarly, an earlier study reported that the glass transition (Tg) temperature of pure dextran molecule and iron-oxide dextran-coated magnetic nanoparticle was approximately 102 °C. Furthermore, the same study demonstrated that the onset of dextran degradation was observed at 50 °C when dextran was adsorbed on magnetic nanoparticles^38^. In this study, the DSS nanoparticles showed a unique endothermic peak at 165.77 °C in 12.46 min. An earlier report suggested that praziquantel–dextran hydrogel showed an endothermic peak at 138.7 °C^39^.

### X-ray diffractometer (XRD) analysis of DSS nanoparticles

The XRD studies are used to characterize the discrete crystalline nano particle structure. In the present study, XRD analysis at 2*θ* showed the presence of pure DSS nanoparticles based on from specific diffraction peaks at 21.56°, 33.37°, 38.73°, 47.17°, 52.96°, and 58.42° (Fig. 6B). The unique peaks at 21.56°, 33.3°, and 47.17° confirmed the unique design of the nanoparticles. An earlier study suggested that dextran-coated iron oxide nanoparticles had a characteristic peak with maximum intensity at 35.79°^40^. In line with the earlier reported work, the present also showed a distinct, prominent peak at 33.3°, indicating the formation of DSS nanoparticles. These results demonstrate the absence of impurities in the DSS nanoparticles, and confirmed that the DSS nanoparticles were nanocrystals with a discrete nature. An earlier report showed peaks at 311°, 400°, 422°, 511°, 440° representing the crystal structure of iron-oxide dextran nanoparticles^38^.

### Antibacterial effects of DSS nanoparticles

The DSS nanoparticles manifested a promising and broad spectrum of antibacterial effects against selected Gram-positive and Gram-negative bacteria (Table 2). The MICs of the DSS nanoparticles against the screened organisms ranged from 150 to 250 μg/mL, depending on the organism. Therefore, in this study, a nanoparticle concentration of 250 μg/mL was used in the antibacterial spectrum experiments. The study determined the potential antibacterial effect of DSS nanoparticles on the screened organisms (Table 2). Surface charge and size of nanoparticles are essential factors for targeting bacterial cells, and they determine degree of penetration into the bacterial cell. Studies have shown that anionic particles are safer than cationic nanoparticles that cause damage to the bacterial cell wall or cell membrane^41^.

**Table 2:** Antibacterial study of dextran sulfate sodium (DSS) nanoparticles of batch 3.

Organisms	Concentration (CFU^a^/mL)	MIC (µg/mL)	Zone of inhibition (mm)	
			DSS nanoparticles	Ciprofloxacin (50 µg/mL)
*Bacillus subtilis*	2 × 10^−5^	150	23.5 ± 2	25.5 ± 1.2
*Staphylococcus aureus*	4 × 10^−5^	200	20.8 ± 1	25 ± 0.8***
*Streptococcus pyogenes*	4 × 10^−3^	250	24.6 ± 0.8	25.6 ± 0.9
*Escherichia coli*	3 × 10^−5^	150	26 ± 1.2	27 ± 1.6
*Pseudomonas aeruginosa*	2 × 10^−3^	200	21.8 ± 1	22.8 ± 1
*Klebsiella pneumoniae*	2 × 10^−4^	250	22 ± 2	24.16 ± 1.3
*Proteus vulgaris*	3 × 10^−3^	250	20.5 ± 0.9	22.16 ± 1.3*

Each value is the mean of 6 batches with standard deviation.

The statistical analyses were done using the Prism 9, Graph Pad Instat software system, USA.

The test values were compared with the standard drug values using Dunnet’s post hoc test.

***Extremely significant at *p* ≤ 0.001when compared to DSS nanoparticles; *significant at *p* ≤ 0.01 when compared to DSS nanoparticles.

^a^*CFU* colony forming unit.

**Figure 7:**
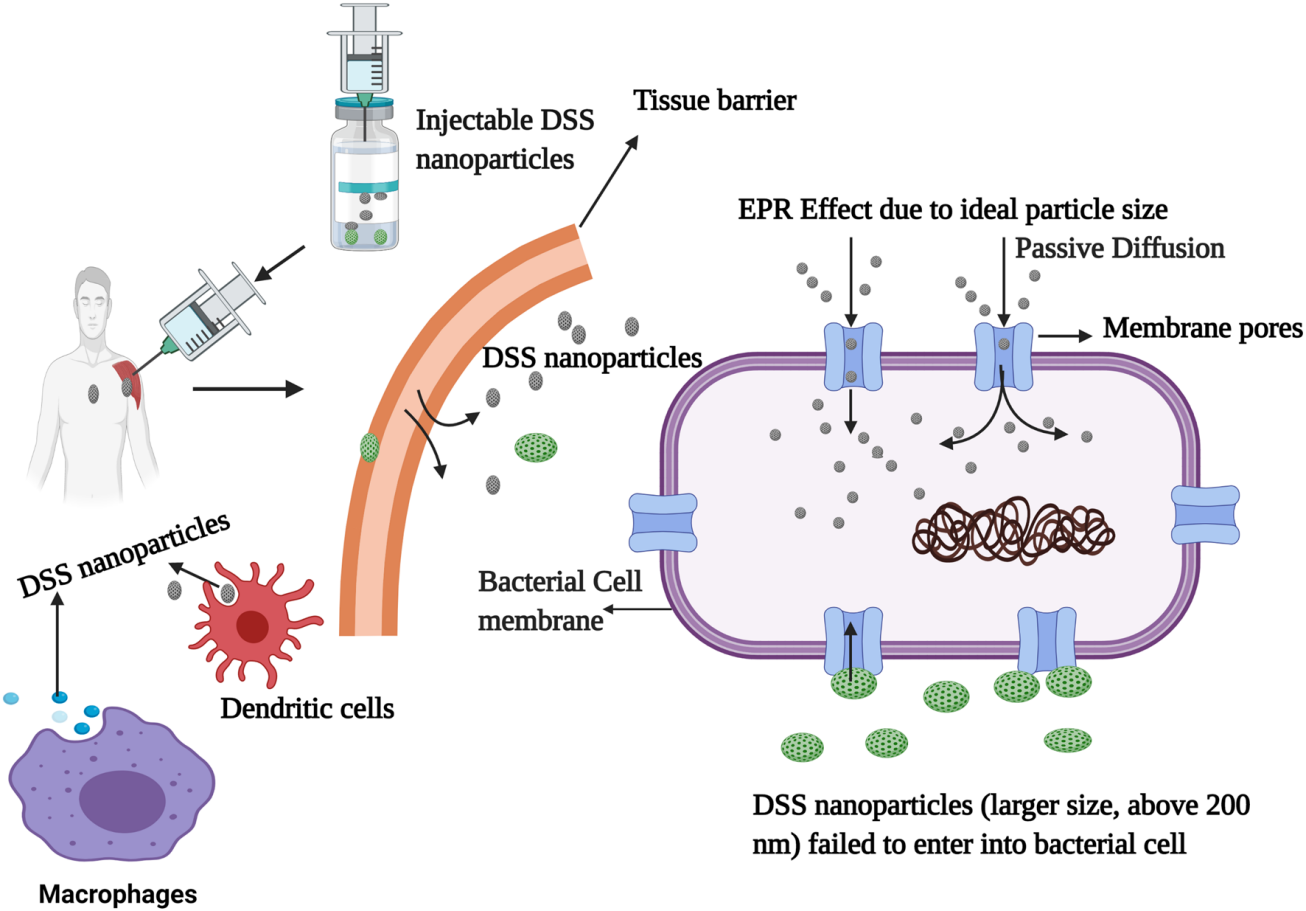
Diagrammatic representation of enhanced permeability and retention (EPR). During passive diffusion through the bacterial cell membrane, the DSS nanoparticle size impacts the bacterial cell diffusion. Passive diffusion can be feasible when the particle size is less than 200 nm. This figure was created with BioRender.com, Bio Render, Canada.

In 2016, a report demonstrated that dextran sulfate silver nanoparticles produced potent antimicrobial activity against *Staphylococcus aureus*, *Bacillus cereus*, *Bacillus luteus*, *Bacillus subtilis*, *Listeria monocytogenes*, *Escherichia coli, Pseudomonas aeruginosa*, *Klebsiella pneumoniae*, *Proteus vulgaris*, and *Candida albicans*^42^. Chemically-modified dextran has been reported to be a potent antibacterial agent, specifically against *P. aeruginosa* and *S. aureus*^19^. An earlier study reported the antibacterial efficacy of an immobilized dextran-curcumin conjugate against the Gram-positive bacteria *L. monocytogenes* and *S. aureus*; and Gram-negative bacteria *E. coli* and *Salmonella typhimurium*. The study reported that Gram-positive bacteria were more sensitive to the dextran-curcumin conjugate than Gram-negative bacteria^43^. In the present study, DSS nanoparticles exhibited a spectrum of activity against Gram-positive and Gram-negative bacteria. The antibacterial effect was highest against *E. coli*, followed by *S. pyogenes, B. subtilis, K. pneumoniae, P. aeruginosa, S. aureus,* and *P. vulgaris*, in that order. The spectrum of antibacterial effect of DSS was almost equivalent to that of the standard drug ciprofloxacin. An earlier report indicated that the nanoparticles of sizes 50–200 nm are ideal for cellular uptake because the particles can easily pass through the cell membrane through passive targeting^44^. In this study the particle size was below 100 nm and thereby achieving the pharmacokinetic properties required for injectable formulations (Fig. 7). The size of nanoparticles influences clearance from the human body^44, 45^. Furthermore, the injection of DSS nanoparticles attracts innate immune cells since their sizes are below 100 nm. This is an added advantage in the treatment of infectious diseases.

## Conclusion

In this study, DSS nanoparticles were developed using gelation technique by forming hydrogen bond. The formulation was successful in terms of physicochemical parameters, and it elicited a good spectrum of antibacterial effects. These findings suggest that DSS nanoparticles are promising antibacterial agents that can be developed as novel therapeutic formulations for combating bacterial infections.
